# COVID‐19‐related lockdowns and changes in overweight and obesity, movement behaviours, diet quality, and health‐related quality of life among regional Australian primary school children: A repeat cross‐sectional study

**DOI:** 10.1111/ijpo.13195

**Published:** 2024-12-18

**Authors:** Claudia Strugnell, Cadeyrn J. Gaskin, Denise Becker, Liliana Orellana, Michelle Jackson, Monique Hillenaar, Jillian Whelan, Andrew D. Brown, Vicki Brown, Colin Bell, Josh Hayward, Lena D. Stephens, Hayley Jensen, Izzy Gribben, Lee Coller, Diana Tatlock, Elizabeth Lehman, Steven Allender

**Affiliations:** ^1^ Institute for Physical Activity and Nutrition Deakin University Geelong Victoria Australia; ^2^ Global Centre for Preventative Health and Nutrition (GLOBE), Institute for Health Transformation Deakin University Geelong Victoria Australia; ^3^ Biostatistics Unit, Faculty of Health Deakin University Geelong Victoria Australia; ^4^ Deakin Health Economics, Institute for Health Transformation Deakin University Geelong Victoria Australia; ^5^ Department of General Practice, School of Public Health and Preventive Medicine Monash University Melbourne Victoria Australia

**Keywords:** children, COVID‐19, obesity, physical activity, sedentary behaviour, sleep

## Abstract

**Background:**

During the coronavirus disease 2019 (COVID‐19) pandemic, the Australian state of Victoria (in particular, its capital, Melbourne) experienced some of the longest lockdowns in the world.

**Objective:**

This repeated cross‐sectional study examined changes between March to June 2019 (pre‐pandemic) and April to August 2022 (6 to 11 months following pandemic‐related lockdowns) in overweight and obesity prevalence, physical activity, sedentary behaviour, sleep, diet quality, and health‐related quality of life (HRQoL) among primary school children in north‐east Victoria, Australia.

**Methods:**

Height and weight were measured for Grade 2, 4, and 6 students in 2019 (3889 children) and 2022 (1816 children). Grade 4 and 6 students self‐reported on their movement behaviours, diet quality, and HRQoL.

**Results:**

Participation declined among schools (2019:56%, 2022:34%) and students (2019:87%, 2022:75%). Compared to children in 2019, children in 2022 had a higher prevalence of overweight and obesity; were less likely to have met guidelines for moderate‐to‐vigorous physical activity, recreational screen time, and vegetable consumption; had higher intakes of takeaway food, energy‐dense nutrient‐poor snacks, and sugar‐sweetened beverages; and had lower HRQoL.

**Conclusion:**

Children's health‐related behaviours and outcomes seemed not to have returned to pre‐pandemic levels 6 to 11 months after the final lockdowns lifted for their communities. Continued monitoring and interventions targeting the drivers of childhood obesity are urgently needed.

## 1 INTRODUCTION

Poorer child health and well‐being are documented, unintended consequences of the public health measures introduced to limit the spread of the coronavirus disease 2019 (COVID‐19).^1^, ^2^, ^3^, ^4^, ^5^, ^6^ International evidence shows that school closures, lockdowns, and other restrictions of movement and socializing were associated with increased unhealthy behaviours^1^, ^7^, ^8^, ^9^, ^10^ and adverse health outcomes^1^, ^2^, ^3^, ^4^, ^5^, ^6^ among children. Lockdowns and other pandemic‐related public health measures were associated with less physical activity,^1^, ^7^, ^8^, ^9^ more screen time,^1^, ^7^, ^8^, ^10^ altered sleep patterns (e.g. later bedtimes and wake‐up times),^1^, ^7^ increased overall food consumption (particularly of unhealthy foods),^1^ and decreased fruit and vegetable intake.^1^ Some evidence suggests the extent of behaviour changes were associated with the length and severity of lockdowns.^7^ In Spain, for example, where strict lockdowns were enforced, physical activity reduced from a mean of 154 min/day to 63 min/day among children aged 8–16 years, and screen time increased from 4.3 to 6.1 mean hours/day.^11^ In jurisdictions with less severe restrictions, physical activity levels remained the same (e.g. Western Australia^12^) or increased (e.g. Germany^13^). The pandemic‐related public health measures have also been associated with increases in body mass index *z*‐scores (BMIz) (mean difference 0.13; 95% confidence interval [CI]: 0.10, 0.17; 20 studies), prevalence of obesity (pooled risk difference 0.02; 95% CI: 0.01, 0.03; 12 studies),^4^ poorer mental health,^1^, ^2^, ^3^, ^6^ and diminished health‐related quality of life (HRQoL)^6^ among children and adolescents.

Following the cessation of the pandemic‐related public health measures, researchers have begun exploring whether child behaviour and health and well‐being outcomes have returned to pre‐pandemic levels. Evidence gathered so far shows that children's physical activity remained below pre‐pandemic levels,^14^, ^15^ and sedentary behaviour continued to be elevated,^14^, ^15^ in the months following the cessation of lockdown restrictions. The Active‐6 study in the United Kingdom (UK), for example, showed that children aged 10–11 years were engaged in 7.7 min/day less moderate‐to‐vigorous physical activity (MVPA) on weekdays (6.9 min/day less on weekend days) and were sedentary for 25.4 more minutes per weekday (14.0 min/day more during weekend days) after the lockdowns than a comparative sample prior to the pandemic.^14^ A limited amount of evidence also suggests that adverse changes in weight‐related outcomes,^16^, ^17^, ^18^ mental health (e.g. symptoms of anxiety and depression),^6^ and HRQoL^6^ persisted after lockdowns and other restrictions had ended.^6^ Data from England's National Child Measurement Programme, for example, showed that the obesity rate among 10‐–11‐year‐old children in 2022/23 (22.7%) decreased from its peak in 2020/21 (25.5%), but remained above the 2019/20 mark (21.0%).^18^ Continued poor outcomes compared with pre‐pandemic levels could signal a need for public health responses.

Evidence on demographic disparities in child behavioural changes and outcomes is mixed. Some studies have shown that pandemic‐related public health measures may have widened gender and socioeconomic inequalities.^16^, ^18^, ^19^, ^20^ Following the UK lockdowns, for example, the gender gap in the activity levels of 10–11 year olds increased, with girls less likely to be highly active and more likely to be sedentary and inactive compared to boys.^20^ Analyses of UK data also showed the socioeconomic gap widened in favour of degree‐educated households.^20^ In a German study, there was an inverse association between socioeconomic status (measured at the school level) and BMI standard deviation scores.^16^ Among 10‐–11‐year‐old children in England's National Child Measurement Programme, the deprivation gap for obesity between the least‐ and most‐deprived deciles widened from 15.6% points in 2019/20 to 17.1% points in 2022/23.^18^ In contrast, other research has shown that changes in child behaviours and outcomes were unrelated,^14^, ^15^ or minimally related,^21^ to gender^14^, ^15^, ^21^ and socioeconomic status.^14^

During the first 2 years of the COVID‐19 pandemic, the Australian state of Victoria (in particular, its capital Melbourne) experienced some of the longest periods of lockdown in the world.^22^ In metropolitan Melbourne, six lockdowns were enforced between 31 March 2020 and 21 October 2021, lasting 263 days in total.^23^, ^24^ Regional Victoria had, depending on the area, seven or eight lockdowns lasting between 124 and 186 days. Lockdowns were sporadic, of different time periods, adjusted frequently, and consisted of stay‐at‐home orders, travel restrictions (up to 5 km from home), and, for metropolitan Melbourne, curfews (9 pm to 5 am, nightly).^23^, ^24^, ^25^ The stay‐at‐home orders limited citizens to five reasons for leaving home: to obtain food and supplies, to receive or provide care, to exercise outdoors, to undertake authorized work, and to get vaccinated at the nearest location (when vaccinations became available).^23^, ^24^ Schools were closed other than to children of essential workers and vulnerable children, playgrounds and skate parks were closed, and outdoor exercise in public was limited to 2 h per day during most of the lockdown periods.^23^, ^24^

Using data from our Reflexive Evidence and Systems interventions to Prevention Obesity and Non‐communicable Disease (RESPOND) trial,^26^ we investigated whether overweight and obesity prevalence, movement behaviours (physical activity, sedentary behaviour, and sleep), diet quality, and HRQoL changed between 2019 and 2022 among primary school children living in communities subjected to severe and lengthy pandemic‐related public health measures between March 2020 and as long as October 2021. We also explored potential gender and socioeconomic inequalities, on which the current evidence is equivocal.^14^, ^15^, ^16^, ^18^, ^19^, ^20^, ^21^

## 2 METHODS

### 2.1 Design

This is a secondary analysis of data collected from communities as part of the RESPOND trial^26^ and nearby non‐randomized communities. RESPOND was a 4‐year step‐wedge cluster‐randomized trial of a community‐led systems science approach^27^ to addressing the complex and dynamic causes of childhood obesity. Ten local government areas (LGAs) in north‐east Victoria, Australia, were randomly allocated to begin the intervention in July 2019 (Step 1, five LGAs) or July 2021 (Step 2, five LGAs). Two additional (non‐randomized) LGAs were monitored during the same time period. Child‐level data were collected at baseline (March to June 2019), with further data collection points planned for Year 2 (2021) and Year 4 (2023). The pandemic‐related restrictions meant that the delivery of the intervention to Step 1 LGA was disrupted and the roll out of the intervention to the Step 2 LGA was paused. In addition, data could not be collected in 2021 as planned and instead occurred from April to August 2022. Data were collected from school children in Grades 2, 4, and 6 (aged approximately 8, 10, and 12, respectively) using an opt‐out approach to student recruitment. Ethics approval for the trial was received from Deakin University Human Research Ethics Committee (HREC 2018–381) and relevant education bodies (Victorian Government Department of Education and Training, 2019_003943; Catholic Archdiocese of Melbourne, Catholic Education Melbourne, 2019–0872; and Catholic Archdiocese of Sandhurst, 24 May 2019). A detailed description of the trial's methods is available from the published protocol.^26^ Data were collected from the non‐randomized LGAs using the same methods as for the LGAs in the trial.

### 2.2 Setting

The LGAs are located in the Goulburn Valley and Ovens Murray regions of north‐east Victoria, Australia. Collectively, the 12 LGAs (including the two non‐randomized LGAs) had approximately 28 000 children aged 5–12 years, with 12 371 across Grades 2, 4, and 6 at the time of the 2016 Australian Census.^28^ All primary schools (government, independent, and Catholic) were invited to participate, with 91/163 (55.8%) schools participating at baseline in 2019 and 54/161 (33.5%) in 2022. The change in the number of schools available to be invited (163–161) was due to two schools merging and one school closing permanently.

### 2.3 Pandemic‐related lockdowns

**FIGURE 1:**
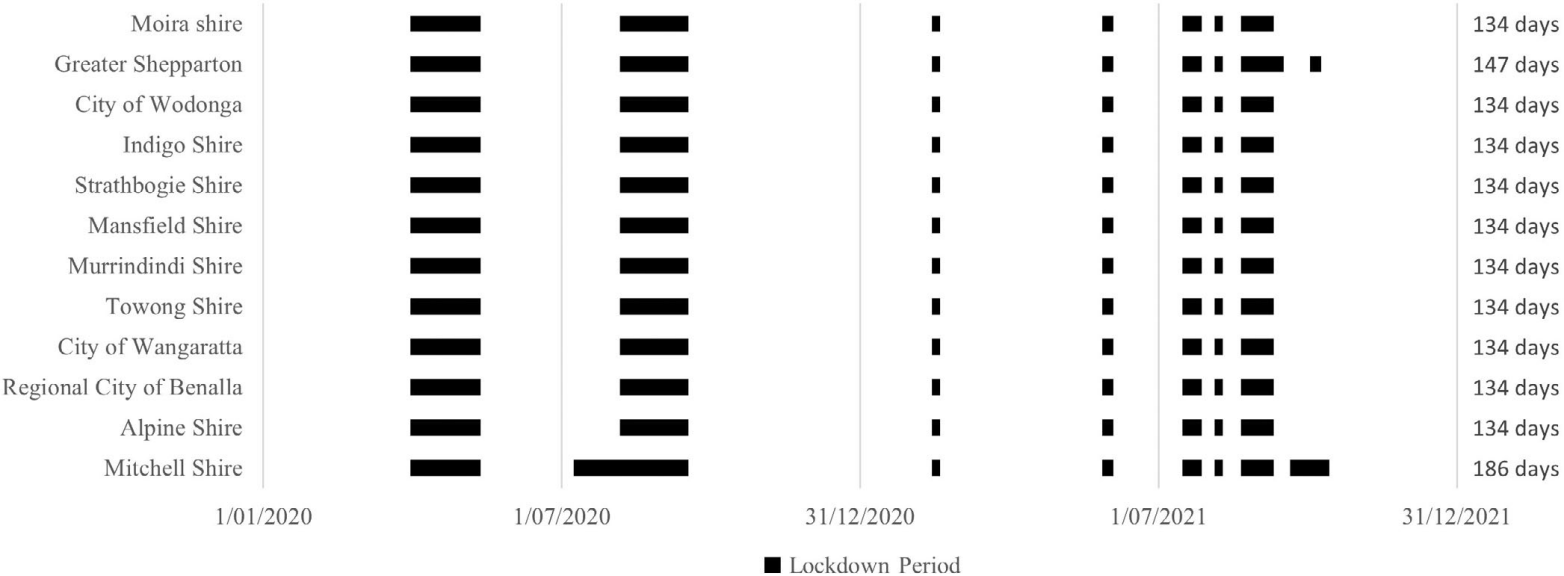
Timeline of State Government imposed lockdown periods of the 12 local government areas.

The pandemic‐related lockdowns affected all 12 LGAs. For ten LGAs, there were seven lockdown periods between 31 March 2020 and 9 September 2021, lasting 134 days in total (see Figure 1). One LGA had eight lockdowns between 31 March 2020 and 8 October 2021, lasting a total of 147 days. One LGA had eight lockdowns between 31 March 2020 and 13 October 2021, lasting 186 days in total.

### 2.4 Participants

Children enrolled at participating schools were eligible to participate in the trial if they were in Grades 2, 4, or 6, and had not opted out (verbally or in‐writing) on the day of data collection. There were no exclusion criteria.

### 2.5 Measures

School characteristics were obtained from government sources. Trained researchers attended the classrooms and communal areas (e.g. school halls) of Grade 2, 4, and 6 students at participating schools. They measured each child's height and weight in private measuring booths using standardized protocols. The trained researchers also supervised children in Grades 4 and 6 as they used electronic tablets to self‐report their movement behaviours (physical activity, sedentary behaviour, and sleep), diet quality, and HRQoL.

#### 2.5.1 School characteristics

Socioeconomic status at the school level was measured with the Index of Community Socio‐Educational Advantage (ICSEA).^29^ ICSEA is a measure of a school's socio‐educational advantage and incorporates both school factors (geographic location and proportion of indigenous students) and student factors (parents' occupations and parents' education). The average ICSEA value is set at 1000, with higher values indicative of greater socio‐educational advantage of students attending the school. ICSEA values were obtained from the *My School* website (www.myschool.edu.au).^30^ Schools were identified as government, Catholic, or independent. School remoteness was classified using the Australian Statistical Geography Standard (ASGS) Edition 3 Remoteness Areas.^31^ Australia is divided into five Remoteness Areas based on relative geographic access to services: major cities, inner regional, outer regional, remote, and very remote.

#### 2.5.2 Demographics

Each child reported their gender and date of birth, which were cross‐checked with school class lists. Grade 4 and 6 students reported their Aboriginal and/or Torres Strait Islander background and language spoken at home.

#### 2.5.3 Body mass index

Trained researchers measured children's heights to the nearest 0.1 cm using a portable stadiometer (Charder HM‐200P Portstad, Charder Electronic Co Ltd., Taichung City, Taiwan) and weights to the nearest 0.1 kg using an electronic weight scale (A&D Precision Scale UC‐321; A7D Medical, San Jose, CA). Two measurements were taken unless values varied by ≥0.5 cm or >0.05 kg, in which case a third measure was taken. The average of all measurements was used in analyses. The World Health Organization's child growth reference^32^ was used to generate age‐ and sex‐specific BMIz and to categorize overweight and obesity.

#### 2.5.4 Movement behaviours

MVPA and recreational screen time for each of the past 7 days, as well as usual method of travelling to and from school during the past 7 days, were measured using three items from the physical activity and sedentary behaviour module of the Core Indicators and Measures of Youth Health.^33^ Usual bedtimes on school nights and wake‐up times on school mornings were reported with using items based on questions used in previous research.^34^ Responses were used to assess their adherence to Australia's 24‐h Movement Guidelines for MVPA (≥60 min/day), recreational screen time (≤2 h per day), and sleep (9–11 h per night).^35^

#### 2.5.5 Diet quality

Fruit and vegetable intakes were assessed using two items modified from the Child Nutrition Questionnaire.^36^ Consumption of energy‐dense nutrient‐poor snacks (e.g. lollies and biscuits), sugar‐sweetened beverages (e.g. fruit juice, soda, and flavoured milk), and takeaway food were reported using 14 items from the Food, Health, and Choices questionnaire (FHC‐Q).^37^ Water consumption was measured using one item from a dietary questionnaire.^38^ Responses were used to assess adherence to the Australian Dietary Guidelines for vegetables (≥5 serves of vegetables for girls aged 9–13 years and boys aged 9–1 years, and ≥5.5 serves for boys aged 12–13 years) and fruit (≥2 serves).^39^ In the absence of recommendations, cut‐points were created for takeaway food (≤1 per fortnight), energy‐dense nutrient‐poor snacks (<1 per day), and sugar‐sweetened beverages (<1 per day).

#### 2.5.6 Health‐related quality of life

HRQoL was measured using the child self‐report (ages 8–12 years) version of the Paediatric Quality of Life Inventory (PedsQL 4.0) Generic Core Scale.^40^, ^41^, ^42^ This instrument has 23 items that assess four domains of functioning: physical (8 items), emotional (5 items), school (5 items), and social (5 items). Domain scores are summed to provide an overall total health‐related quality of life score and summary scores for physical health (generated from items in the physical functioning domain) and psychosocial health (generated from items in the emotional, social, and school functioning domains). Responses for each of the items were reverse‐scored and linearly transformed to a 100‐point scale, with higher scores indicative of better quality of life.

### 2.6 Data analysis

Characteristics of the sample of schools and children recruited in 2019 and 2022 were compared using t tests, chi‐squared tests, or Fisher's exact tests, as appropriate. Changes between 2019 and 2022 in mean HRQoL scores were estimated using linear mixed models. Changes between 2019 and 2022 in the prevalence of overweight/obesity and meeting movement and dietary guidelines (binary outcomes) were estimated using logistic mixed models. All models included school as a random effect. LGA was not incorporated as a higher‐level random effect because its contribution to variance was negligible after accounting for school clustering. For each outcome, a model was fitted that included year (2019, 2022), gender, the interaction gender×year, child grade, school ICSEA (<1000/≥1000), school rurality (mayor city or inner regional/outer regional) and intervention received (1 if a school participating in 2022 belonged to an LGA allocated to intervention, 0 otherwise) as fixed effects. These factors were included to adjust for potential imbalances in children or schools participating in both waves and for any intervention effect. For each outcome, the following contrasts, alongside 95% CIs and *p*‐values, are reported: mean change (continuous outcomes) or prevalence change (binary outcomes) between 2022 and 2019 for the whole sample and by gender; and the difference in change between genders. To investigate potential moderation of socio‐educational school level (ICSEA <1000 vs. ≥1000) on changes between 2019 and 2022, we fitted models including ICSEA, year and the interaction ICSEA×year, child gender, grade, school rurality, and intervention. For each outcome, we report mean change (continuous outcomes) or prevalence change (binary outcomes) between 2022 and 2019 within ICSEA level and difference in change between ICSEA levels. We did not adjust for multiplicity of outcomes. Due to the repeat cross‐sectional design, there were no dropouts; the number of missing observations for outcome variables was negligible for children participating in each wave. Factors used to adjust for confounding did not have missing values because they were school level variables (ICSEA, school rurality) or data documented in school records (gender, grade). All analyses were performed in STATA (version 18.0).

## 3 RESULTS

Child response rates and reasons for non‐participation in 2019 and 2022 are presented in Table 1. Between 2019 and 2022, there were increases in opting‐out (13%–25%) and absenteeism (9%–17%) across all three grade levels.

**TABLE 1:** Number of children who participated, opted out, and were absent during data collection among those enrolled in participating schools by grade and year.

	Grade 2, 4 and 6				Grade 2				Grade 4				Grade 6			
	2019		2022		2019		2022		2019		2022		2019		2022	
Enrolled in participating schools	4922		2945		1619		975		1708		1010		1595		960	
Absent	466	9%	503	17%	121	7%	164	17%	15	1%	183	18%	194	12%	156	16%
Eligible	4456		2442		1498		811		1693		827		1401		804	
Opted out (*n*, % of eligible)	566	13%	622	25%	210	14%	187	23%	181	11%	214	26%	175	12%	221	27%
Participated (*n*, % of eligible)	3890	87%	1820	75%	1288	86%	624	77%	1376	81%	613	74%	1226	88%	583	73%

Characteristics of the children and schools are provided in Table 2. Compared to children in 2019, children in 2022 were slightly older and more likely to have spoken a language other than English at home. More schools participated in 2019 (*n* = 91) than 2022 (*n* = 52), with 47 schools involved in both years.

**TABLE 2:** Child and school characteristics in 2019 and 2022.

	2019			2022			
Child characteristics	N	*n*	%	N	*n*	%	*p* ^a^
Gender^b^							
Male	3889	1959	50.4	1816	923	50.8	0.5877
Female	3889	1917	49.3	1816	875	48.2	
Undisclosed/missing	3889	13	0.3	1816	18	1.0	
Grade							
2	3889	1288	33.1	1816	624	34.4	0.3793
4	3889	1376	35.4	1816	609	33.5	
6	3889	1225	31.5	1816	583	32.1	
Aboriginal and/or Torres Strait Islander	2092	212	10.1	1006	113	11.2	0.3484
Language other than English spoken at home	2569	209	8.1	1165	156	13.4	<0.0001
	N	M	SD	N	M	SD	*p* ^c^
Age (years)	3888	9.79	1.7	1816	9.91	1.7	<0.0001
School characteristics	N	n	%	N	n	%	*p* ^a^
Socioeconomic status (school ICSEA)							
≥1000	91	35	38.5	52	19	336.5	0.8592
<1000	91	56	61.5	52	33	63.5	
School type							
Government	91	84	92.3	52	47	90.4	0.8899
Catholic	91	4	4.4	52	2	3.8	
Independent	91	3	3.3	52	3	5.8	
School remoteness							
Major cities	91	1	1.1	52	1	1.9	0.9999
Inner regional	91	76	83.5	52	44	84.6	
Outer regional	91	14	15.4	52	7	13.5	
School participation							
One year only (2019 or 2022)	91	44	48.4	52	5	9.6	<0.0001
Both years (2019 and 2022)	91	47	51.6	52	47	90.4	
Intervention							
Yes	91	32	35.2	52	15	28.8	0.5642
No	91	35	38.5	52	19	36.5	
Non‐randomized	91	24	26.4	52	18	34.6	
	N	M	SD	N	M	SD	*p* ^c^
Socioeconomic status (school ICSEA)	91	980.84	55.65	52	981.00	62.09	0.9870

Abbreviation: ICSEA, Index of Community Socio‐Educational Advantage.

^a^ Fisher's exact test.

^b^ Test excluding undisclosed and missing data.

^c^ *T*‐test.

The estimated changes in outcomes between 2019 and 2022 are presented in Table 3. Compared to children in 2019, children in 2022 had a significantly higher prevalence of overweight and obesity (+4.0% points [pp]; 95% CI: 0.7, 7.4). Children in 2022 were less likely to be meeting guidelines for MVPA over 5 days/week (−10.1 pp.; 95% CI: −14.4, −5.9) and 7 days/week (−9.1 pp.; 95% CI: −12.5, −5.6), and guidelines for recreational screen time over 5 days/week (−6.0 pp.; 95% CI: −10.0, −2.1) and 7 days/week (−9.7 pp.; 95% CI: −14.0, −5.5). Children in 2022 were significantly less likely to be meeting guidelines for vegetable consumption (−3.5 pp.; 95% CI: −6.5, −0.5) and to have infrequent consumption of takeaway food (≤1/fortnight; −14.5 pp.; 95% CI: −18.4, −10.6), energy‐dense nutrient‐poor snacks (<1/day; −5.7 pp.; 95% CI: −9.9, −1.6), and sugar‐sweetened beverages (<1/day; −5.1 pp.; 95% CI: −9.3, −1.0). Children in 2022 had significantly lower scores across all HRQoL domains: physical (−5.2 points; 95% CI: −6.5, −3.9), school (−6.7 points; 95% CI: −8.3, −5.2), emotional (−7.4 points; 95% CI: −9.2, −5.6), and social (−4.7 points; 95% CI: −6.4, −3.0).

**TABLE 3:** Estimated changes from 2019 to 2022 in prevalence of overweight/obesity, meeting movement behaviour guidelines, diet quality, and in mean health‐related quality of life scores.

	2019			2022			Δ (2022–2019)	
	N	*n*	% (95% CI)	N	*n*	% (95% CI)	Estimate^a^ (95% CI)	*p* ^a^
Overweight/obesity^b^	3702	1306	35.1 (33.1, 37.1)	1711	644	39.1 (36.2, 42.1)	4.0 (0.7, 7.4)	0.0192
Meeting movement behaviour guidelines:								
MVPA (≥ 1 hr/ day, 5 days/wk)	2581	1056	43.7 (40.3, 47.1)	1173	369	33.6 (29.4, 37.8)	−10.1 (−14.4, −5.9)	<0.0001
MVPA (≥ 1 hr/ day, 7 days/wk)	2581	595	24.1 (21.6, 26.7)	1173	172	15.1 (12.2, 17.9)	−9.1 (−12.5, −5.6)	<0.0001
Recreational screen time (≤ 2 hrs/day, 5 days/wk)	2578	1845	72.6 (70.0, 75.1)	1176	777	66.5 (62.8, 70.3)	−6.0 (−10.0, −2.1)	0.0029
Recreational screen time (≤ 2 hrs/day, 7 days/wk)	2578	1375	55.0 (52.3, 57.8)	1176	516	45.3 (41.4, 49.2)	−9.7 (−14.0, −5.5)	<0.0001
Active transport (to and/or from school)	2584	888	31.4 (27.1, 35.7)	1177	398	29.1 (24.3, 33.8)	−2.4 (−6.2, 1.5)	0.2286
Sleep (9–11 hrs/day)	2341	1718	73.3 (71.2, 75.4)	1083	785	72.3 (69.1, 75.5)	−1.0 (−4.8, 2.8)	0.6063
Diet quality								
Vegetables (≥5 serves/day, ≥5.5 for boys 12+)	2581	416	16.4 (14.5, 18.2)	1173	156	12.9 (10.5, 15.2)	−3.5 (−6.5, −0.5)	0.0213
Fruit (≥ 2 serves/day)	2582	1881	72.7 (70.6, 74.8)	1177	861	72.7 (69.6, 75.8)	0.0 (−3.7, 3.7)	0.9990
Takeaway (≤ 1/fortnight)	2582	1531	60.3 (58.3, 62.4)	1175	567	45.9 (42.6, 49.1)	−14.5 (−18.4, −10.6)	<0.0001
Energy‐dense nutrient‐poor snacks (<1/day)	2581	941	38.2 (35.3, 41.0)	1177	378	32.4 (28.7, 36.2)	−5.7 (−9.9, −1.6)	0.0066
Sugar‐sweetened beverages (<1/day)	2582	1445	57.1 (54.6, 59.6)	1177	618	52.0 (48.3, 55.6)	−5.1 (−9.3, −1.0)	0.0159
Water (≥ 5 glasses/day)	2582	1419	55.6 (53.4, 57.8)	1176	641	53.5 (50.2, 56.9)	−2.1 (−6.2, 2.0)	0.3133
	N		Mean (95% CI)	N		Mean (95% CI)	Estimate^a^ (95% CI)	*P* ^a^
Health‐related quality of life								
Total	2560		75.7 (74.9, 76.6)	1170		69.8 (68.6, 71.0)	−5.9 (−7.2, −4.6)	<0.0001
Physical	2552		81.7 (80.8, 82.5)	1170		76.4 (75.3, 77.6)	−5.2 (−6.5, −3.9)	<0.0001
Psychosocial	2559		72.7 (71.7, 73.7)	1168		66.3 (65.0, 67.7)	−6.3 (−7.8, −4.9)	<0.0001
School	2560		73.0 (72.0, 74.0)	1169		66.3 (64.9, 67.7)	−6.7 (−8.3, −5.2)	<0.0001
Emotional	2550		67.3 (66.1, 68.5)	1169		59.9 (58.2, 61.5)	−7.4 (−9.2, −5.6)	<0.0001
Social	2551		77.5 (76.4, 78.7)	1167		72.8 (71.3, 74.4)	−4.7 (−6.4, −3.0)	<0.0001

Abbreviations: ICSEA, Index of Community Socio‐Educational Advantage; MVPA, moderate‐to‐vigorous physical activity; wk., week.

^a^ Estimates from logistic (binary outcomes) and linear (continuous outcomes) mixed models with a random effect (school) and fixed effects (year, gender, year×gender interaction, grade, school ICSEA, school rurality, and intervention).

^b^ Grades 2, 4, and 6 (all other outcomes Grades 4 and 6 only).

Changes between 2019 and 2022 for boys and girls and differences in changes between the genders are provided in Table S1. Changes over time were significantly different between boys and girls for fruit consumption and school functioning. For boys, there was a non‐significant increase in the prevalence of meeting guidelines of fruit consumption (3.9 pp.; 95% CI: −0.9, 8.7), and, for girls, there was a non‐significant decrease in this prevalence (−4.1 pp.; 95% CI: −8.9, 0.6), with the difference in changes between 2019 and 2022 significantly favouring boys (8.1 pp.; 95% CI: 1.9, 14.2). Significant decreases in HRQoL school functioning scores between 2019 and 2022 were evident for both boys (−5.2 points; 95% CI: −7.1, −3.2) and girls (−8.4 points; 95% CI: −10.4, −6.4), but the change was significantly smaller in boys than girls (3.2 points; 95% CI: 0.8, 5.6).

There was no evidence that socio‐educational school level (ICSEA below and above mean) moderated the observed changes in outcomes between 2019 and 2022 (Table S2).

## 4 DISCUSSION

Our findings potentially illustrate the collision of the COVID‐19 pandemic with the obesity epidemic.^43^ Six to 11 months after the last lockdown, the enduring effects of these public health measures seemed evident in the RESPOND data for several child behaviours and outcomes. Children participating in 2022 had a higher prevalence of overweight and obesity than their counterparts in 2019. They were less likely to be meeting guidelines for physical activity and recreational screen time. They were less likely to be meeting guidelines for vegetable consumption and had higher intakes of takeaway food, energy‐dense nutrient‐poor snacks, and sugar‐sweetened beverages. Children in 2022 also had lower HRQoL across all domains (physical, school, emotional, and social). Widening gender and socioeconomic inequalities were not evident.Of particular concern was the higher prevalence of overweight and obesity in 2022. In many high‐income countries, the trends in child body mass index (BMI) had plateaued at high levels.^44^ Pandemic‐related public health measures may have inadvertently reignited this upward trend. Consistent with international evidence,^16^, ^17^, ^18^ the prevalence of overweight and obesity among children in regional Victoria was higher after the lockdowns had ceased compared to pre‐pandemic levels. There is a possibility that data in some of these studies,^16^, ^17^ and our own data, were collected too soon (approximately 6 to 12 months after lockdowns) to detect a return to pre‐pandemic levels. Data from England's National Child Measurement Programme showed that the obesity rate among 10‐–11‐year olds continued to decline from its peak in 2020/21, but remained above pre‐pandemic levels up to 2 years later (in 2022/23).^18^

Differences in behaviour disfavouring children in 2022—less physical activity, more screen time, higher intakes of energy‐dense nutrient‐poor snacks, sugar‐sweetened beverages, and potentially unhealthy takeaway foods—may provide insights into the increases in overweight and obesity. Substantial differences in activity levels disfavouring children in 2022 seemed evident, with a 9.1% point difference in children meeting guidelines for MVPA over 7 days per week, and a 9.7% point difference in children meeting guidelines for recreational screen time over 7 days per week. The findings on children's activity levels add to the emerging international evidence that physical activity and sedentary behaviour had not returned to pre‐pandemic levels in the months following the cessation of restrictions.^14^, ^15^ Extended periods of restrictions on movement seem to have had enduring effects on physical activity and sedentary behaviour, at least 6–11 months after the lifting of lockdowns. The lengthy lockdowns may have created the conditions for enduring behaviour change. For example, more permissive parental attitudes towards screen time during lockdowns (e.g. encouraging screen time to connect with friends and to promote quiet environments when working from home^7^) may have made it challenging for them to reinstate stricter screen time rules after lockdowns ended. Diets were unhealthier in 2022, with children less likely to be meeting guidelines for vegetable consumption (−3.5% points) and to have low intakes of takeaway food (≤1/fortnight; −14.5% points), energy‐dense nutrient‐poor snacks (<1/day; −5.7% points), and sugar‐sweetened beverages (<1/day; −5.1% points). Adding to the international^1^ and Australian^45^ evidence that children consumed more food during the pandemic‐related restrictions, our findings show that children were consuming poorer quality food 6–11 months after the public health measures had lifted than children prior to the pandemic. Parents from many countries have reported that, during the pandemic, families ate more diverse foods and home‐cooked meals, alongside overeating and increased snacking of energy‐dense nutrient‐poor foods due to parents adopting more permissive attitudes towards food.^46^ The findings from our study may suggest that these more relaxed attitudes towards food may have continued post‐lockdowns.

Self‐reported HRQoL (all domains) was lower for children assessed approximately 6–11 months after the lifting of restrictions than those assessed before the pandemic. This finding is consistent with international evidence showing that declines in HRQoL during the pandemic persisted after restrictions lifted.^6^ Using criteria from the developers of the PedsQL 4.0 Generic Core Scale,^41^ the differences between children in the total HRQoL score and the psychosocial HRQoL domain are clinically important. Although the restrictions had lifted, certain stressors (e.g. adjusting to in‐person schooling, concern over infection, and the unpredictability of the situation) may have underpinned these findings.

Although there were differences favouring boys over girls for meeting guidelines for fruit consumption and school functioning, the apparent changes over time reduced the differences between boys and girls rather than creating or magnifying inequalities. The percentages of boys and girls meeting guidelines for fruit consumption were wider apart in 2019 (68.2% vs. 77.5%) than 2022 (72.1% vs. 73.4%). Similarly, the social functioning scores of boys and girls were wider apart in 2019 (70.6 vs. 75.6) than 2022 (65.4 vs. 67.2). In both years, the mean percentages of girls meeting guidelines and their mean social functioning scores were higher than those for boys.

### 4.1 Strengths and limitations

There are several key strengths of this study. First, collecting data 6–11 months after the lockdowns ended potentially enabled the capturing of rebound effects as children began to establish post‐lockdown routines. This timing contrasts with the previous studies in which data were collected soon after lockdowns ceased,^15^, ^16^ which was potentially too soon for children to have re‐established pre‐pandemic behaviours. Second, the response rates were high (87% in 2019 and 75% in 2022) using an opt‐out approach, compared with typical 30%–60% response rates when opt‐in approaches are used.^47^ Third, trained researchers objectively measured height and weight rather than relying on self‐ or proxy‐reporting. Fourth, we documented the activity levels of children living in regional areas. A regional perspectivity is important because children living in regional areas have higher rates of overweight and obesity compared to their metropolitan counterparts, and examining changes over time is important to ensure inequities are not widening.^48^ Fifth, we present findings on children's diet quality, which has received less attention in the literature than activity levels.

The study has limitations. First, the repeated cross‐sectional design involved a different sample of schools and children participating at each data collection point, which might explain at least part of the observed differences. Between 2019 and 2022, there were decreases in the participation rates of schools (60%–32%) and children (87.3%–74.5%). The drop in participation among schools seemed due to the burden on Victorian schools to provide education for students at a time of regular and, at times, prolonged school closures to manage COVID‐19. Child absenteeism and opting out was higher in 2022, which may have been due to COVID‐19 infections and an increased wariness among parents, carers, and children associated with public health measures (e.g. testing for COVID‐19, vaccination mandates). Although we controlled for the available school and children characteristics, residual confounding due to factors other than the lockdowns may have produced these findings. For example, there may have been differences between 2019 and 2022 in unmeasured child characterizes, such as family‐level socioeconomic status and family home environments (e.g. more permissive parenting). Second, the differences observed could not be attributed to the COVID‐19 lockdowns as the study did not have a direct measure of their impact. Evidence from a qualitative study, however, shows that lockdowns did affect rural food supply.^49^ In addition, the restrictions in place during the lockdowns limited opportunities for physical activity and socialization. Third, self‐reported (rather than objective) measures of diet, physical activity, and sedentary behaviour were used. Fourth, the generalizability of the findings is limited. There was considerable heterogeneity in lockdown lengths and settings (e.g. stay‐at‐home orders, travel restrictions, and curfews) across countries, across Australian states, and within Australian states. The study, however, provides some evidence of the potential consequences of prolonged lockdowns on children's behaviours and health outcomes.

### 4.2 Implications for policy and practice

The findings from this study demonstrate a need for public policy action and continued funding of routine population‐level monitoring of child health‐related behaviours and outcomes, chronic disease prevention programs, and efforts to improve child mental health. Routine monitoring (as occurs with England's National Child Measurement Programme) would provide the data needed to support the case for, and evaluation of, public health measures designed to change child health‐related behaviours and to enhance child outcomes. With child health‐related behaviours and outcomes having not returned to pre‐pandemic levels, there would seem to be merit in designing and implementing local‐level interventions to improve behaviours and outcomes. One of the targets of such interventions would need to be children's mental health. These findings reinforce the importance of Australia's National Children's Mental Health and Wellbeing Strategy for promoting the well‐being of children and supporting those experiencing challenges with their mental health.^50^

## 5 CONCLUSION

In Victoria, public health measures during the COVID‐19 pandemic appear to have had adverse unintended consequences. Data from our repeat cross‐sectional sample of children from regional Victoria before and after the restrictions seem to suggest that more children were living with overweight and obesity and that they were less likely to have met guidelines for MVPA, recreational screen time, and vegetable consumption; had higher intakes of takeaway food, energy‐dense nutrient‐poor snacks, and sugar‐sweetened beverages; and had lower HRQoL across all domains (physical, school, social, and emotional). These differences were irrespective of gender and the socioeconomic position of the schools that children attended.

## AUTHOR CONTRIBUTIONS

S.A., C.S., L.O., C.B., J.H., and V.B. developed the overall study design. C.S., M.J., M.H., J.W., J.H., L.D.S., H.J., I.G., L.C., D.T., E.L. collected the data. D.B. and L.O. analysed the data. C.S., C.J.G., D.B., L.O., M.J., M.H., C.B., A.B., V.B. and S.A. interpreted the data. C.J.G. performed the literature search and generated the figure. C.S. and C.J.G. led the writing of the manuscript. All authors read and approved the final manuscript.

## FUNDING INFORMATION

Funding and/or in‐kind support for this project was received from the Australian National Medical and Health Research Council (NHMRC) (GNT 1151572), Victorian Department of Education, Beechworth Health Service, Yarrawonga Health, Lower Hume Primary Care Partnership, Upper Hume Primary Care Partnership, the Department of Health Victoria, Numurkah District Health Service, Central Hume Primary Care Partnership, Nexus Primary Health, VicHealth, Goulburn Valley Primary Care Partnership, Gateway Health. SA is supported by NHMRC Ideas grant GNT2002234. The contents of this publication are solely the responsibility of the authors and do not reflect the views of the NHMRC, the Victorian Department of Education or the Department of Health Victoria.

## CONFLICT OF INTEREST STATEMENT

The authors have no conflicts of interest to declare.

## Supporting information


**Table S1.** Estimated changes from 2019 to 2022 within boys and girls in prevalence of overweight/obesity, meeting movement behaviour guidelines, diet quality, and in mean health‐related quality of life scores.
**Table S2**. Estimated changes from 2019 to 2022 within children attending schools with ICSEA≥1000 and ICSEA<1000 in prevalence of overweight/obesity, meeting movement behaviour guidelines, diet quality, and in mean health‐related quality of life scores.

## Data Availability

The datasets used and analysed during the current study are not available for data sharing due to the ethical constraints deployed in an opt‐out approach.
