# Supplementary material for: COVID‐19‐related lockdowns and changes in overweight and obesity, movement behaviours, diet quality, and health‐related quality of life among regional Australian primary school children: A repeat cross‐sectional study
Source: Pediatr Obes. 2024 Dec 18;20(2):e13195. doi: 10.1111/ijpo.13195 (PMC11710946; doi:10.1111/ijpo.13195)
Supplement: Supplementary file 1 — Table S1. Estimated changes from 2019 to 2022 within boys and girls in prevalence of overweight/obesity, meeting movement behaviour guidelines, diet quality, and in mean health‐related quality of life scores. Table S2. Estimated changes from 2019 to 2022 within children attending schools with ICSEA≥1000 and ICSEA<1000 in prevalence of overweight/obesity, meeting movement behaviour guidelines, diet quality, and in mean health‐related quality of life scores. [file IJPO-20-e13195-s001.pdf]

COVID-19-related lockdowns and changes in overweight and obesity, movement behaviours, diet quality, and health-related quality of life among regional Australian primary school children: A repeat cross-sectional study

Claudia Strugnell,[1,2] Cadeyrn J. Gaskin,[2] Denise Becker, [3] Liliana Orellana,[3] Michelle Jackson,[2] Monique Hillenaar,[2] Jillian Whelan,[2] Andrew D. Brown,[2] Vicki Brown,[4] Colin Bell,[2] Josh Hayward,[2] Lena D. Stephens,[2] Hayley Jensen,[2] Izzy Gribben,[2] Lee Coller,[2] Diana Tatlock,[2] Elizabeth Lehman,[5] and Steven Allender[2]

[1] Institute for Physical Activity and Nutrition, Deakin University, Geelong, Australia

[2] Global Centre for Preventative Health and Nutrition (GLOBE), Institute for Health Transformation, Deakin University, Geelong, Australia

[3] Biostatistics Unit, Faculty of Health, Deakin University, Geelong, Australia

[4] Deakin Health Economics, Institute for Health Transformation, Deakin University, Geelong, Australia

[5] Department of General Practice, School of Public Health and Preventive Medicine, Monash University, Melbourne, Australia

Corresponding Author

Dr Claudia Strugnell, Institute for Physical Activity and Nutrition, Deakin University Waterfront Campus, Geelong, Victoria, Australia 3220. Tel: +61 3 5227 8483 Email: claudia.strugnell@deakin.edu.au

Table S1. Estimated changes from 2019 to 2022 within boys and girls in prevalence of overweight/obesity, meeting movement behaviour guidelines, diet quality, and in mean health-related quality of life scores.

	Boys								Girls								Difference in Change	
	2019			2022			Δ (2022-2019)		2019			2022			Δ (2022-2019)		Boys (2022-2019) - Girls (2022-2019)	
	N	n	% (95% CI)	N	n	% (95% CI)	Estimate[1] (95% CI)	*P*[1]	N	n	% (95% CI)	N	n	% (95% CI)	Estimate[1] (95% CI)	*P*[1]	Difference[1] (95% CI)	*P*[1]
Overweight/obesity[2]	1890	661	34.8 (32.3, 37.3)	889	353	41.3 (37.6, 45.1)	6.6 (2.2, 10.9)	0.003	1812	645	35.4 (32.9, 38.0)	822	291	36.8 (33.0, 40.6)	1.4 (-3.0, 5.8)	0.5446	5.2 (-0.3, 10.7)	0.0661
Meeting movement behaviour guidelines																		
MVPA (≥ 1 hr/ day, 5 days/wk)	1312	623	49.7 (45.8, 53.6)	618	238	40.9 (35.7, 46.1)	-8.8 (-14.2, -3.3)	0.0017	1268	433	37.3 (33.4, 41.1)	539	124	25.7 (20.9, 30.4)	-11.6 (-16.7, -6.4)	<0.0001	2.8 (-3.6, 9.2)	0.3897
MVPA (≥ 1 hr/ day, 7 days/wk)	1312	374	29.2 (25.9, 32.5)	618	110	18.4 (14.6, 22.3)	-10.8 (-15.5, -6.1)	<0.0001	1268	221	18.6 (15.9, 21.4)	539	58	11.4 (8.3, 14.6)	-7.2 (-11.1, -3.3)	0.0003	-3.6 (-8.8, 1.7)	0.1813
Recreational screen time (≤ 2 hrs/day, 5 days/wk)	1311	883	68.3 (65.1, 71.6)	619	380	61.6 (56.9, 66.4)	-6.7 (-11.9, -1.5)	0.0121	1266	961	77.1 (74.2, 79.9)	541	385	71.8 (67.3, 76.2)	-5.3 (-10.2, -0.4)	0.0325	-1.4 (-7.7, 4.9)	0.6644
Recreational screen time (≤ 2 hrs/day, 5 days/wk)	1311	649	51.0 (47.7, 54.4)	619	244	40.3 (35.6, 45.0)	-10.7 (-16.1, -5.3)	0.0001	1266	725	59.3 (56.0, 62.7)	541	267	50.6 (45.6, 55.6)	-8.7 (-14.3, -3.1)	0.0022	-2.0 (-8.8, 4.8)	0.5688
Active transport (to and/or from school)	1314	469	32.3 (27.7, 37.0)	619	230	32.2 (26.7, 37.6)	-0.2 (-5.0, 4.6)	0.9382	1269	419	30.4 (25.9, 35.0)	542	161	25.7 (20.7, 30.8)	-4.7 (-9.4, 0.0)	0.0503	4.5 (-1.1, 10.1)	0.1164
Sleep (9-11 hrs/day)	1169	851	72.7 (69.9, 75.5)	562	407	71.9 (67.8, 76.0)	-0.8 (-5.8, 4.1)	0.7372	1171	867	73.9 (71.2, 76.7)	508	371	72.8 (68.6, 77.0)	-1.2 (-6.2, 3.8)	0.6519	0.3 (-6.1, 6.7)	0.9260
Diet quality																		
Vegetables (≥5 serves/day, ≥5.5 for boys 12+)	1311	198	15.4 (13.1, 17.6)	617	82	12.7 (9.7, 15.6)	-2.7 (-6.4, 1.0)	0.1510	1269	218	17.5 (15.0, 19.9)	541	74	13.1 (10.0, 16.2)	-4.4 (-8.3, -0.4)	0.0299	1.7 (-3.1, 6.4)	0.4914
Fruit (≥ 2 serves/day)	1312	897	68.2 (65.4, 71.0)	619	451	72.1 (68.2, 76.1)	3.9 (-0.9, 8.7)	0.1115	1269	983	77.5 (75.0, 80.0)	542	399	73.4 (69.3, 77.4)	-4.1 (-8.9, 0.6)	0.0873	8.1 (1.9, 14.2)	0.0103
Takeaway (≤ 1/fortnight)	1312	727	56.4 (53.6, 59.2)	617	289	43.9 (39.7, 48.1)	-12.5 (-17.7, -7.4)	<0.0001	1269	804	64.5 (61.8, 67.2)	542	271	48.0 (43.5, 52.4)	-16.5 (-21.7, -11.3)	<0.0001	3.9 (-2.9, 10.7)	0.2558
Energy-dense nutrient-poor snacks (<1/day)	1311	454	36.3 (33.0, 39.7)	619	191	30.7 (26.2, 35.1)	-5.7 (-10.8, -0.6)	0.0292	1269	486	40.1 (36.7, 43.6)	542	182	34.3 (29.5, 39.1)	-5.8 (-11.3, -0.4)	0.0359	0.2 (-6.3, 6.7)	0.9626
Sugar-sweetened beverages (<1/day)	1312	672	52.3 (49.1, 55.4)	618	296	46.7 (42.1, 51.2)	-5.6 (-10.9, -0.3)	0.0401	1269	772	62.2 (59.1, 65.3)	543	315	57.6 (52.9, 62.3)	-4.6 (-10.0, 0.8)	0.0946	-1.0 (-7.8, 5.8)	0.7714
Water (≥ 5 glasses/day)	1312	738	56.9 (54.0, 59.8)	618	350	55.5 (51.2, 59.8)	-1.4 (-6.7, 3.8)	0.5914	1269	680	54.2 (51.3, 57.2)	542	285	51.4 (46.9, 56.0)	-2.8 (-8.3, 2.6)	0.3127	1.4 (-5.5, 8.3)	0.6964
	N		Mean (95% CI)	N		Mean (95% CI)	Estimate[1] (95% CI)	*P*[1]	N		Mean (95% CI)	N		Mean (95% CI)	Estimate[1] (95% CI)	*P*[1]	Difference[1] (95% CI)	*P*[1]
Health-related quality of life																		
Total	1299		75.8 (74.8, 76.9)	616		70.6 (69.2, 72.0)	-5.2 (-6.8, -3.6)	<0.0001	1260		75.6 (74.6, 76.7)	538		69.0 (67.5, 70.4)	-6.6 (-8.3, -5.0)	<0.0001	1.4 (-0.6, 3.4)	0.1676
Physical	1297		82.3 (81.3, 83.3)	616		77.5 (76.0, 78.9)	-4.8 (-6.5, -3.1)	<0.0001	1254		81.0 (80.0, 82.0)	538		75.4 (73.9, 76.9)	-5.6 (-7.4, -3.9)	<0.0001	0.8 (-1.3, 3.0)	0.4529
Psychosocial	1299		72.5 (71.3, 73.7)	614		67.0 (65.4, 68.6)	-5.5 (-7.3, -3.7)	<0.0001	1259		72.9 (71.7, 74.1)	538		65.6 (63.9, 67.3)	-7.3 (-9.2, -5.4)	<0.0001	1.8 (-0.4, 4.1)	0.1067
School	1300		70.6 (69.4, 71.8)	615		65.4 (63.7, 67.1)	-5.2 (-7.1, -3.2)	<0.0001	1259		75.6 (74.4, 76.8)	538		67.2 (65.5, 69.0)	-8.4 (-10.4, -6.4)	<0.0001	3.2 (0.8, 5.6)	0.0093
Emotional	1292		69.4 (68.0, 70.9)	615		62.5 (60.5, 64.5)	-6.9 (-9.1, -4.6)	<0.0001	1257		65.0 (63.6, 66.5)	538		57.0 (54.9, 59.1)	-8.0 (-10.4, -5.7)	<0.0001	1.1 (-1.7, 4.0)	0.4335
Social	1298		77.3 (76.0, 78.6)	614		73.1 (71.2, 75.0)	-4.2 (-6.3, -2.0)	0.0001	1252		77.8 (76.5, 79.2)	537		72.5 (70.6, 74.5)	-5.3 (-7.5, -3.1)	<0.0001	1.1 (-1.6, 3.8)	0.4227

ICSEA, Index of Community Socio-Educational Advantage; MVPA, moderate-to-vigorous physical activity; wk, week.

[1] Estimates from logistic mixed (binary outcomes) and linear (continuous outcomes) models with a random effect (school) and fixed effects (year, gender, year×gender interaction, grade, school ICSEA, school rurality, and intervention).

[2] Grades 2, 4, and 6 (all other outcomes Grades 4 and 6 only).

Table S2. Estimated changes from 2019 to 2022 within children attending schools with ICSEA≥1000 and ICSEA<1000 in prevalence of overweight/obesity, meeting movement behaviour guidelines, diet quality, and in mean health-related quality of life scores.

	ICSEA ≥ 1000								ICSEA < 1000								Difference in Change	
	2019			2022			Δ (2022-2019)		2019			2022			Δ (2022-2019)		≥1000 (2022-2019) - <1000 (2022-2019)	
	N	n	% (95% CI)	N	n	% (95% CI)	Estimate[1] (95% CI)	*P*[1]	N	n	% (95% CI)	N	n	% (95% CI)	Estimate[1] (95% CI)	*P*[1]	Difference[1] (95% CI)	*P*[1]
Overweight/obesity[2]	1316	409	32.2 (29.0, 35.4)	537	156	35.4 (29.7, 41.0)	3.1 (-3.0, 9.3)	0.3172	2386	897	36.5 (34.0, 39.0)	1174	488	40.9 (37.5, 44.3)	4.4 (0.6, 8.2)	0.0229	-1.3 (-8.1, 5.5)	0.7164
Meeting movement behaviour guidelines																		
MVPA (≥ 1 hr/ day, 5 days/wk)	912	410	44.9 (39.6, 50.1)	383	147	36.1 (28.9, 43.3)	-8.8 (-16.3, -1.2)	0.0231	1669	646	43.0 (38.8, 47.2)	790	222	32.5 (27.6, 37.4)	-10.5 (-15.2, -5.8)	<0.0001	1.7 (-6.5, 9.9)	0.6866
MVPA (≥ 1 hr/ day, 7 days/wk)	912	239	25.5 (21.4, 29.7)	383	78	18.4 (12.9, 23.9)	-7.1 (-13.4, -0.8)	0.0262	1669	356	23.1 (19.9, 26.2)	790	94	13.4 (10.3, 16.6)	-9.6 (-13.3, -5.9)	<0.0001	2.5 (-4.3, 9.2)	0.4700
Recreational screen time (≤ 2 hrs/day, 5 days/wk)	909	702	77.0 (73.2, 80.8)	383	287	71.2 (64.4, 77.9)	-5.9 (-13.0, 1.2)	0.1046	1669	1143	70.4 (67.1, 73.6)	793	490	64.1 (59.7, 68.6)	-6.2 (-10.7, -1.7)	0.0071	0.3 (-7.6, 8.2)	0.9336
Recreational screen time (≤ 2 hrs/day, 5 days/wk)	909	539	59.5 (55.1, 63.8)	383	194	46.3 (39.1, 53.5)	-13.2 (-20.9, -5.4)	0.0004	1669	836	52.9 (49.4, 56.4)	793	322	44.2 (39.7, 48.8)	-8.7 (-13.5, -3.9)	0.0004	-4.5 (-13.0, 4.1)	0.3042
Active transport (to and/or from school)	912	312	33.5 (26.8, 40.2)	383	114	28.4 (20.8, 36.1)	-5.0 (-12.1, 2.0)	0.1585	1672	576	30.5 (25.3, 35.6)	794	284	28.9 (23.3, 34.5)	-1.5 (-5.7, 2.7)	0.4725	-3.5 (-11.1, 4.1)	0.3638
Sleep (9-11 hrs/day)	841	626	74.3 (71.0, 77.6)	360	280	78.6 (73.2, 84.0)	4.3 (-1.9, 10.6)	0.1754	1500	1092	72.6 (70.1, 75.1)	723	505	69.9 (66.3, 73.4)	-2.7 (-7.0, 1.5)	0.2099	7.1 (0.0, 14.2)	0.0511
Diet quality																		
Vegetables (≥5 serves/day, ≥5.5 for boys 12+)	912	139	16.0 (13.0, 18.9)	383	55	12.1 (7.9, 16.3)	-3.9 (-8.9, 1.2)	0.1312	1669	277	16.6 (14.3, 19.0)	790	101	13.2 (10.5, 16.0)	-3.4 (-6.8, 0.0)	0.0485	-0.5 (-6.1, 5.1)	0.8651
Fruit (≥ 2 serves/day)	912	695	74.7 (71.3, 78.1)	383	303	75.8 (69.9, 81.7)	1.1 (-5.5, 7.8)	0.7427	1670	1186	71.7 (69.1, 74.4)	794	558	71.5 (67.9, 75.0)	-0.3 (-4.4, 3.8)	0.8994	1.4 (-6.0, 8.7)	0.7125
Takeaway (≤ 1/fortnight)	912	592	64.5 (61.1, 67.8)	382	240	55.5 (49.1, 62.0)	-8.9 (-16.2, -1.6)	0.0162	1670	939	58.1 (55.5, 60.7)	793	327	41.8 (38.1, 45.4)	-16.4 (-20.7, -12.0)	<0.0001	7.4 (-0.6, 15.5)	0.0695
Energy-dense nutrient-poor snacks (<1/day)	912	373	40.6 (36.1, 45.1)	383	169	39.4 (32.2, 46.5)	-1.2 (-9.0, 6.5)	0.7567	1669	568	36.5 (33.0, 40.0)	794	209	29.3 (25.1, 33.6)	-7.2 (-11.7, -2.7)	0.0018	6.0 (-2.5, 14.4)	0.1651
Sugar-sweetened beverages (<1/day)	912	595	64.8 (60.9, 68.7)	383	239	56.1 (49.1, 63.1)	-8.7 (-16.4, -1.1)	0.0245	1670	850	53.3 (50.1, 56.5)	794	379	49.2 (45.0, 53.5)	-4.1 (-8.7, 0.6)	0.0870	-4.7 (-13.0, 3.7)	0.2749
Water (≥ 5 glasses/day)	912	476	52.8 (49.1, 56.4)	382	202	51.3 (44.6, 57.9)	-1.5 (-9.0, 6.1)	0.6997	1670	943	57.1 (54.4, 59.9)	794	439	54.8 (51.0, 58.7)	-2.3 (-6.8, 2.3)	0.3251	0.8 (-7.6, 9.2)	0.8516
	N		Mean (95% CI)	N		Mean (95% CI)	Estimate[1] (95% CI)	*P*[1]	N		Mean (95% CI)	N		Mean (95% CI)	Estimate[1] (95% CI)	*P*[1]	Difference[1] (95% CI)	*P*[1]
Health-related quality of life																		
Total	908		75.9 (74.5, 77.3)	382		70.2 (68.1, 72.4)	-5.7 (-8.0, -3.4)	<0.0001	1652		75.7 (74.6, 76.7)	788		69.7 (68.3, 71.0)	-6.0 (-7.4, -4.6)	<0.0001	0.3 (-2.2, 2.8)	0.8083
Physical	905		81.8 (80.5, 83.1)	382		77.0 (74.8, 79.2)	-4.8 (-7.3, -2.4)	0.0001	1647		81.6 (80.5, 82.6)	788		76.2 (74.9, 77.6)	-5.3 (-6.8, -3.8)	<0.0001	0.5 (-2.2, 3.2)	0.7154
Psychosocial	908		72.7 (71.1, 74.3)	382		66.5 (64.1, 69.0)	-6.2 (-8.8, -3.6)	<0.0001	1651		72.6 (71.4, 73.9)	786		66.3 (64.7, 67.8)	-6.4 (-8.0, -4.8)	<0.0001	0.2 (-2.6, 3.1)	0.8896
School	909		73.2 (71.6, 74.9)	382		66.6 (64.0, 69.2)	-6.6 (-9.4, -3.8)	<0.0001	1651		72.9 (71.6, 74.2)	787		66.2 (64.6, 67.8)	-6.7 (-8.4, -5.0)	<0.0001	0.1 (-2.9, 3.1)	0.9481
Emotional	902		66.2 (64.3, 68.2)	382		59.5 (56.4, 62.5)	-6.8 (-10.0, -3.5)	0.0001	1648		67.8 (66.3, 69.3)	787		60.2 (58.3, 62.1)	-7.6 (-9.6, -5.6)	<0.0001	0.8 (-2.7, 4.4)	0.6472
Social	906		78.5 (76.7, 80.3)	381		74.1 (71.2, 77.0)	-4.4 (-7.5, -1.3)	0.0059	1645		77.0 (75.6, 78.4)	786		72.2 (70.4, 74.0)	-4.8 (-6.7, -2.9)	<0.0001	0.5 (-2.9, 3.9)	0.7801

ICSEA, Index of Community Socio-Educational Advantage; MVPA, moderate-to-vigorous physical activity; wk, week.

[1] Estimates from logistic mixed (binary outcomes) and linear (continuous outcomes) models with a random effect (school) and fixed effects (year, gender, year×gender interaction, grade, school ICSEA, school rurality, and intervention).

[2] Grades 2, 4, and 6 (all other outcomes Grades 4 and 6 only).
